# Prophylactic Intra-Aortic Balloon Pump Implantation Reduces Peri-Interventional Myocardial Injury During High-Risk Percutaneous Coronary Intervention in Patients Presenting with Low Normal Blood Pressure and with Heart Failure

**DOI:** 10.3390/jcm14134796

**Published:** 2025-07-07

**Authors:** Sascha d’Almeida, Stefanie Andreß, Sebastian Weinig, Benjamin Mayer, Wolfgang Rottbauer, Sinisa Markovic, Dominik Buckert

**Affiliations:** 1 Department of Cardiology, Angiology, Pneumology, and Intensive Care Medicine, Ulm University Hospital, 89075 Ulm, Germany; 2 Institute for Epidemiology and Medical Biometry, Ulm University, 89075 Ulm, Germany

**Keywords:** IABP, intra-aortic balloon pump, prophylaxis, high-risk PCI, high-risk Percutaneous Coronary Intervention, myocardial injury, blood pressure, heart failure

## Abstract

**Background**: Intra-aortic balloon pump (IABP) augments coronary perfusion during high-risk percutaneous coronary interventions (PCI). We sought to identify patients who benefited from prophylactic IABP (P-IABP) compared to rescue-IABP (R-IABP). **Methods**: All consecutive non-cardiogenic shock patients undergoing high-risk PCI with IABP support at Ulm University Hospital, Germany, between 2012 and 2020 were grouped based on the timing of IABP insertion in the pre-interventional P-IABP or peri-interventional R-IABP group. We compared the primary endpoint peri-interventional high-sensitivity Troponin T (hsTnT) increase, sought baseline characteristics associated with the endpoint in the R-IABP group, and compared their correlation strengths between the groups. **Results**: Interventional outcomes of 44 patients with P-IABP implantation were compared with those of 15 patients with R-IABP implantation. P-IABP was associated with a lower peri-interventional hsTnT increase (*p* = 0.008, r = 0.390). In the R-IABP group, the presence of ST-segment elevation (*p* = 0.037, r = 0.631), low systolic blood pressure (RRsyst) (*p* = 0.007, r = 0.893 (inverse correlation)), and elevated NT-proBNP levels (*p* < 0.001, r = 0.953) were associated with higher hsTnT increases. HsTnT increase was significantly smaller in the P-IABP group in patients with low RRsyst (IZI = 2.6) and high NT-proBNP levels (IZI = 3.36). Patients with RRsyst < 120 mmHg (*p* = 0.007) and NT-proBNP levels ≥ 900 pg/mL (Cohen’s d = 0.70, respectively 1.17 for ≥5000 pg/mL and 5.01 for ≥10,000 pg/mL) showed lower peri-interventional hsTnT increase when treated with P-IABP compared to R-IABP, while patients with NT-proBNP levels < 900 pg/mL showed a contrary effect (Cohen’s d = −0.90). Cox regression analysis showed that a high peri-interventional hsTnT increase was significantly associated with a shorter survival time (*p* = 0.046). **Conclusions**: P-IABP use in high-risk PCI was associated with reduced peri-interventional myocardial injury, as measured by lower hsTnT increase, which was associated with improved survival in patients with low systolic blood pressure and elevated NT-proBNP levels. Thus, these conditions should be considered for indicating P-IABP.

## 1. Introduction

Intra-aortic balloon pump (IABP) is a counterpulsation device that inflates during diastole to boost coronary perfusion and deflates during systole to decrease afterload, improving myocardial oxygenation and cardiac output and reducing left ventricular workload. IABP is used in conditions like cardiogenic shock, post-myocardial injury, cardiomyopathy, as a bridge to therapy, and in high-risk patients before coronary stenting or cardiac surgery [1]. Compared to other devices such as microaxial left ventricular assist devices (LVAD) and Impella, IABP is simpler, cost-effective, easy to manage, and generally has lower complication rates [2]. This was demonstrated by a recent study of patients undergoing percutaneous coronary intervention (PCI) for acute myocardial infarction complicated by cardiogenic shock, which found that intravascular LVAD use was associated with increased short-term and one-year risk of mortality, bleeding, renal replacement therapy, and cost compared with IABP [3,4]. Furthermore, IABP use is associated with fewer major bleeding events and lower in-hospital costs than Impella use. Notably, these lower complication rates with IABP do not come at the cost of increased mortality compared to Impella [5]. This was confirmed by the PROTECT II trial, which showed no significant difference in the results between IABP and Impella in high-risk PCI patients [6]. These findings of reduced bleeding complication rates and comparable mortality rates for IABP in comparison to Impella were also corroborated by a study on patients with acute myocardial infarction complicated by cardiogenic shock [7]. These features make IABP the most common mechanical support for a failing heart [8]. Importantly, given the rather modest hemodynamic effect compared to advanced mechanical circulatory support devices [2] and the drastic consequences of progression to cardiogenic shock [9,10], timely IABP insertion is crucial. Early implantation of percutaneous mechanical circulatory support devices before PCI in cases of acute myocardial infarction complicated by cardiogenic shock has been associated with better survival outcomes [11]. Specifically, early microaxial LVAD implantation reduces early and midterm all-cause mortality compared to later use as a bailout strategy [12]. However, evidence on the indications for prophylactic IABP (P-IABP) implantation in patients with non-cardiogenic shock is limited. Therefore, this study sought to identify criteria that indicate the benefit of early P-IABP use in hemodynamically stable patients undergoing high-risk PCI.

## 2. Methods

### 2.1. Study Design

In this retrospective, monocentric cohort study, all consecutive patients who underwent high-risk PCI with IAPB support at the University Heart Center, Ulm, Germany, between 2012 and 2020 were included. High-risk PCI regarding the use of mechanical circulatory support was defined according to the criteria set forth by the Society for Cardiovascular Angiography and Interventions (SCAI)/American College of Cardiology (ACC)/Heart Failure Society of America (HFSA)/Society of Thoracic Surgeons (STS) 2015 expert consensus statement criteria based on coronary lesion, patient, and clinical presentation. Accordingly, interventions of the last remaining vessel, left main, bifurcation disease, ostial stenoses, saphenous vein grafts, heavily calcified lesions, and chronic total occlusions or complex three-vessel disease, with a focus on the area of myocardium at risk during procedural ischemia [6,13,14,15] in at-risk patients, were included. Risk factors included multivessel or left main disease, a history of myocardial infarction, impaired left ventricular function, symptoms of heart failure, older age, and comorbidities such as chronic kidney disease, diabetes mellitus, peripheral artery disease, and acute ischemia [15]. Patients underwent PCI for the emergent or elective treatment of coronary occlusion, including partial or total stenosis. The presence of cardiogenic shock before or at the time of IABP insertion was an exclusion criterion, as the effect of IABP support in this patient population has already been investigated without any benefit being demonstrated [16]. Hemodynamic instability or cardiogenic shock was defined as a systolic blood pressure below 90 mmHg or low-output failure requiring the administration of vasoactive agents, including catecholamines, vasopressors, or inotropes, according to the definition of the Cardiogenic Shock Initiative (CSI) [10]. Patients with hemodynamic support devices, such as Impella or ECMO, were also excluded, as they could disguise the need for catecholamines. Patients were grouped according to the time of insertion of the IABP. The P-IABP group included patients who received the device electively prior to PCI at the discretion of the interventionalist. The rescue-IABP group (R-IABP) comprised patients in whom insertion was performed during the procedure due to unexpected circulatory deterioration. Circulatory deterioration was defined as worsening of the cardiocirculatory state with vital parameters falling below the normal values, specifically a drop in blood pressure or increase in heart rate, but without fulfilling the criteria for shock as described above, thus corresponding to a pre-shock state.

The study was approved by the local ethics committee (ethics application no. 40.19) and adheres to the Declaration of Helsinki.

### 2.2. Data Collection, Laboratory Analysis, and Follow-Up

Demographic, clinical, and laboratory data at baseline, during the intervention, and at follow-up were extracted from our patient management system. Clinical assessment and focused cardiovascular examinations, such as 12-lead ECG, transthoracic echocardiography, and drawing of blood samples, were performed at admission and again during the hospital stay. Left ventricular systolic function (LVSF or left ventricular ejection fraction, LVEF) was measured during the index procedure by ventriculography in two projections (30 RAO/0 cranial and 40 LAO/0 cranial) and afterwards again by transthoracic echocardiography (EPIQ 7, Koninklijke Philips N.V., Eindhoven, The Netherlands) and categorized as normal (1), mildly impaired (2), moderately impaired (3) and severely impaired (4) according to guideline-specific recommendations [17]. High-sensitivity Troponin T (hsTnT) and N-terminal pro-natriuretic peptide (NT-proBNP) levels were measured from blood samples (ElectroChemiLumineszenz ImmunoAssay “ECLIA” Roche, Cobas 8000).

### 2.3. Endpoints

The primary endpoint was the peri-interventional increase in hsTnT levels during PCI, along with its predictors. We examined the impact of early pre-interventional prophylactic (P-)IABP implantation compared to that of later peri-interventional bailout or rescue (R-)IABP implantation on the peri-interventional increase in hsTnT. We sought criteria to identify patients at risk for a large peri-interventional hsTnT increase in advance in the R-IABP group. The correlation strength of the identified predictors was compared between the groups to identify criteria for the presence of which peri-interventional hsTnT increase could be reduced through a P-IABP strategy compared to an R-IABP strategy.

To examine the clinical implications of our primary endpoint results, mortality was assessed as a secondary endpoint. We calculated its association with peri-interventional hsTnT increase and identified predictors.

### 2.4. Cut Point Testing for NT-proBNP Levels

Due to the variability of NT-proBNP levels depending on clinical context, patient characteristics, and previous medical history, we analyzed different cut-off points to identify the most appropriate for our broad cohort of patients undergoing high-risk PCI. Candidate cut-off points were NT-proBNP levels of ≥900 pg/mL, which is the cut-off point for the diagnosis of heart failure in patients in the present age group of 70 to 75 years [18], and ≥5000 and ≥10,000 pg/mL according to previously published cut-offs for acute severe and acute very severe heart failure [19,20]. For each candidate cut-off point, the groups were divided according to the NT-proBNP level at admission into subgroups with levels (1) below and (2) equal to or above this threshold. To quantify the effect of P-IABP compared to R-IABP in patients who exceeded a certain cut-off point and those who did not, we calculated the effect size of P-IABP compared to R-IABP on the primary endpoint, peri-interventional hsTnT increase, for the resulting subgroups.

### 2.5. Statistical Analysis

Continuous variables are presented as mean ± standard deviation or median along with interquartile range (IQR) as appropriate. The Kolmogorov-Smirnov test was used to assess the normal distribution of continuous parameters. If a metric variable was not normally distributed at any time of measurement, all values were presented as medians together with the IQR. Categorical variables are described as numbers and percentages. Student’s *t*-test, Mann-Whitney U-test, or Chi^2^ test were used to compare variables between groups, where appropriate. Point-biserial correlation was performed to examine the effect of P-IABP on the primary endpoint of peri-interventional hsTnT increase. Pearson or Spearman’s rank correlation analyses were performed to identify baseline characteristics associated with the primary endpoint in the R-IABP group. Potential confounding effects due to differences in baseline characteristics were excluded using partial correlation analysis, controlling for the variables with significant differences, as appropriate. Correlation coefficients were provided along with the 95% Confidence Interval (CI). The correlation strength of the identified baseline characteristics with a significant association with the peri-interventional increase in hsTnT in the R-IABP group was compared with the corresponding correlation coefficient in the P-IAPB group using R to Z transformation. An IZI value > 1.96 was considered to indicate a statistically significant difference. To address potential confounding by unmeasured variables, we performed a sensitivity analysis using the partial correlation method to simulate the presence of an unmeasured confounder (Z) that could influence both the baseline characteristics and clinical outcomes. We assumed varying strengths of correlation between Z and both variables: mild confounding, rXZ = rYZ = 0.2; moderate confounding, rXZ = rYZ = 0.4; and strong confounding, rXZ = rYZ = 0.6. Cohen’s d was used to assess the effect size. A d ≥ 0.2 was defined as a small effect, a d ≥ 0.4 as a moderate effect, and a d ≥ 0.8 as a strong effect. Cox regression analysis was used to assess the effect of peri-interventional hsTnT increase on survival time. The dependent variable was time to event, defined as the number of days from the index event to death or the last follow-up. Patients who had not experienced death by the end of follow-up were right-censored on the date of the last known survival. Parameters with a *p*-value < 0.05 were considered statistically significant. Statistical analysis was performed using SPSS Statistics 20.0 and 29 software (SPSS Inc., Chicago, IL, IBM in New York, and Version 2022, IBM, Armonk, NY, USA). Due to the explorative nature of this study, all results of the statistical tests must be interpreted as generating hypotheses.

## 3. Results

### 3.1. Study Population

During the study period between 2012 and 2020, 300 consecutive patients underwent PCI with IAPB support at our tertiary care center at Ulm University Hospital. After excluding patients with cardiogenic shock, 59 patients were included in the study. Of these, 44 patients received pre-interventional P-IAPB and 15 underwent peri-interventional R-IABP implantation. All patients included were classified as SCAI A.

### 3.2. Baseline Characteristics

In both groups, the mean age was slightly above 70 years (P-IABP, 74.02 ± 9.56 years vs. R-IBP, 70.93 ± 12.57 years, *p* = 0.33), and most patients were male (P-IABP, 68.2% vs. R-IABP, 80%, *p* = 0.38) without significant difference. In the P-IABP group, fewer patients were admitted as emergencies (P-IABP, 77.3% vs. R-IABP group, 100%; *p* = 0.04). Additionally, patients in the P-IABP group had less frequent ST-segment elevations (P-IABP, 22.7%; R-IABP, 60.0%; *p* = 0.01). Beyond that, no significant differences were observed in the patients’ baseline characteristics. In particular, cardiac function parameters, including LVEF grade, which was mostly severely reduced, frequency of severe aortic valve stenosis and mitral regurgitation, left main and ostial right coronary artery (RCA) stenosis, and NT-proBNP levels, were comparable between the groups. Coronary lesion complexity, as estimated by the Syntax score, was also comparable. The proportion of patients with a history of interventional or surgical coronary revascularization was similar.

No patient in the P-IABP group had an IABP inserted before coronary angiography. All patients in the P-IABP group received IABP after angiographic assessment and before PCI. Most PCI procedures in both groups were performed ad hoc after diagnostic coronary angiography. A small number of eight patients underwent staged PCI, meaning the PCI was scheduled for a later session after diagnostic angiography. All eight patients were treated with P-IABP (P-IABP, 18.2% vs R-IABP, 0%, *p* = 0.003). The main indication for coronary angiography in both groups was acute coronary syndrome, while acute and chronic heart failure were more frequent in the P-IABP group (*p* = 0.002).

The baseline characteristics are presented in Table 1.

### 3.3. Procedural Data

There were no significant intergroup differences in the procedural data. In particular, the length of the procedure, initial TIMI flow, and number of stents placed were similar. The most common primary vessel of intervention in both groups was the left main artery (P-IAPB, 56%,; R-IABP, 30.8%), followed by the left anterior descending artery (LAD) (P-IABP, 24.4; R-IABP, 38.4%) with no significant difference between the groups (*p* = 0.43). The complication rates were also similar. One patient in the P-IABP group experienced cardiogenic shock during the procedure due to coronary dissection with pericardial effusion. This was managed by pericardial drainage and autotransfusion. Three patients experienced femoral complications at the IABP access site, including two with pseudoaneurysms and one with a large hematoma. One patient had a pseudoaneurysm of the radial artery following access for coronary angiography and PCI. All these complications affected patients in the P-IABP group (P-IABP vs. R-IABP, *p* = 0.304 and *p* = 0.564, respectively). The procedural data for both groups are shown in Table 2.

### 3.4. Course of hsTnT over the Procedure for P-/R-IABP

**Figure 1:**
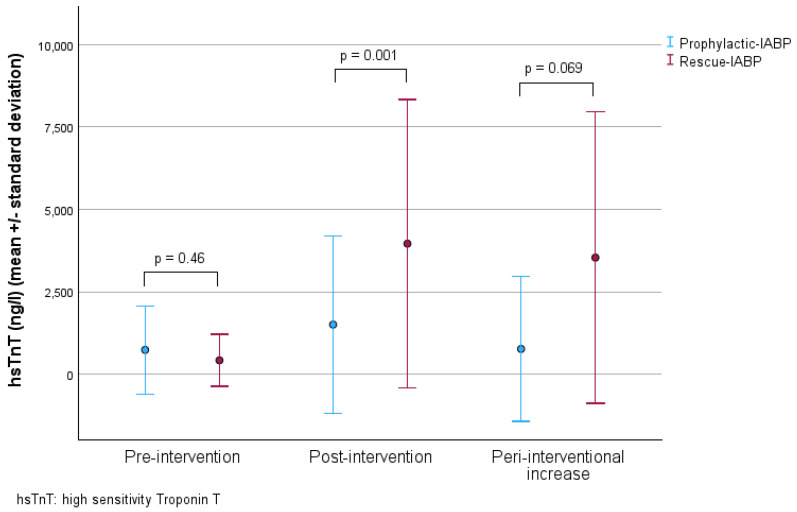
Dot plot with error bars of hsTnT levels at baseline, post-procedure, and peri-interventional increase in the P- and R-IABP group, each compared with Student’s *t*-test.

While hsTnT levels before PCI were comparable at admission (P-IABP: 738.76 ± 1339.61 vs. R-IABP: 423.0 ± 787.22, *p* = 0.46), post-procedure levels were lower in the P-IABP group (P-IABP: 1411.81 ng/L ± 2451.34 ng/L vs. R-IABP: 4557.15 ng/L ± 4306.72 ng/L, *p* = 0.001). This was accompanied by a strong trend toward a lower increase in hsTnT levels in the P-IABP group (P-IABP 767.85 ± 2199.05 ng/L vs. R-IABP 3538.91 ± 4421.74 ng/L, *p* = 0.069). Point-biserial correlation demonstrated that P-IABP was associated with a lower peri-interventional hsTnT increase (*p* = 0.008, r = 0.390). HsTnT levels at different time points of measurement, along with their peri-interventional course, are shown in Table 3 and illustrated in Figure 1.

### 3.5. Predictors of Peri-Interventional hsTnT Increase and Their Effect in P-IABP vs. R-IABP

Pearson correlation analysis for the association of baseline variables with the primary endpoint peri-interventional hsTnT increase in the R-IABP group found that the presence of ST-segment elevation (*p* = 0.037, r = 0.631), low systolic blood pressure (RRsyst) (*p* = 0.007, r = 0.893), and elevated NT-proBNP levels (*p* < 0.001, r = 0.953) were significantly associated with and strongly correlated with greater increases in hsTnT. There was no significant correlation between the baseline characteristics sex, Syntax score, left main stenosis, proximal RCA stenosis, LVEF, severe aortic valve stenosis, severe mitral regurgitation, systolic pulmonary artery pressure (sPAP), left bundle branch block, right bundle branch block, ST-segment depression, T-wave inversion, hsTnT, creatinine, creatine kinase (CK), and history of coronary revascularization such as stenting and coronary artery bypass graft (Table 4). In the P-IABP group, no significant correlation or moderate or strong correlation was observed for any of the variables that were correlated with the primary endpoint in the R-IABP group (ST-segment elevation *p* = 0.074, r = 0.310; RRsyst *p* = 0.856, r = −0.036; NT-proBNP *p* = 0.762, r = 0.051) (Table 5). Comparison of the strength of correlation of these variables with peri-interventional hsTnT increase between the R-IABP group and P-IABP group revealed a significant difference for the criteria of low RRsyst (IZI = 2.6) and high NT-proBNP levels (IZI = 3.36), but not for ST-segment elevation (IZI = 1.07).

A sensitivity analysis was conducted to adjust for potential unmeasured confounders. After adjusting for hypothetical confounding by an unmeasured confounder Z, the correlation between each of the identified baseline predictors, RRsyst and NT-proBNP, and the endpoint peri-interventional hsTnT increase remained strong. For RRsyst, the correlation remained at −0.889 for low (rXZ = rYZ = 0.2), −0.872 for moderate (rXZ = rYZ = 0.4), and −0.833 for strong (rXZ = rYZ = 0.6) confounding. For NT-proBNP, the correlation remained at 0.951 for low (rXZ = rYZ = 0.2), 0.944 for moderate (rXZ = rYZ = 0.4), and 0.927 for strong (rXZ = rYZ = 0.6) confounding.

### 3.6. Normal Blood Pressure vs. Hypertension

**Figure 2:**
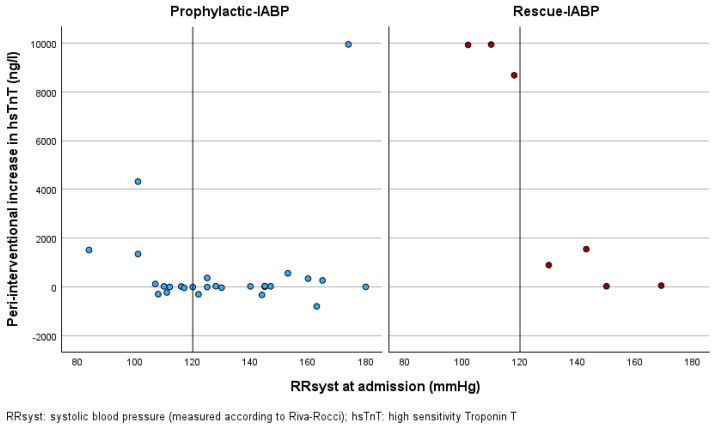
Scatter plot of the correlation between RRsyst and the primary endpoint peri-interventional hsTnT increase in the P-IABP and R-IABP group, with the dividing line marking the border between normal and hypertensive blood pressure values (RRsyst 120 mmHg).

**Figure 3:**
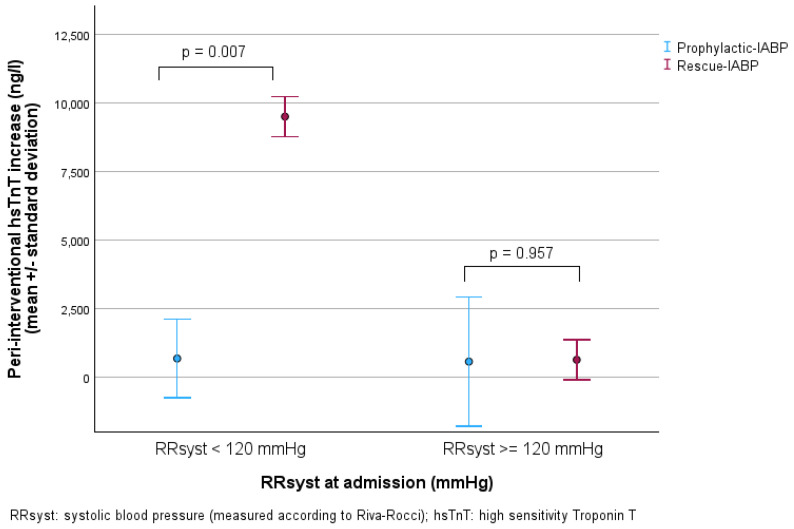
Dot plot with error bars of the primary endpoint peri-interventional hsTnT increase in patients with normal (<120 mmHg) and hypertensive (≥120 mmHg) RRsyst in the P-IABP and R-IABP group, each compared with the Student’s *t*-test.

The correlation between RRsyst and peri-interventional hsTnT increase for both groups is shown in Figure 2. To further evaluate the effect of P-IABP compared to R-IABP depending on blood pressure levels, we compared the subgroups of patients with normal blood pressure values and those with hypertension. Normal blood pressure was defined as RRsyst < 120 mmHg, according to the guidelines [21]. Among patients with normal blood pressure, those receiving P-IABP had a significantly lower peri-interventional increase in hsTnT levels compared to patients who were treated with R-IABP (682.50 ± 1425.85 ng/L vs. 9514 ± 723.18 ng/L, *p* = 0.007). Patients with hypertension in the P-IABP and R-IABP group showed similar peri-interventional increases in hsTnT levels (569.50 ± 2358.50 ng/L vs. 636.25 ± 731.54 ng/L, *p* = 0.957) (Figure 3).

### 3.7. Testing for NT-proBNP Cut Points

**Figure 4:**
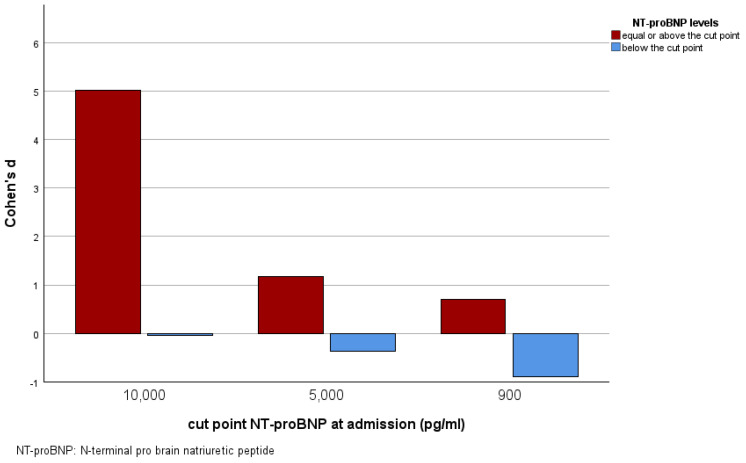
Bar chart of the effect size measured by Cohen’s d in the P-IABP group compared to the R-IABP group according to NT-proBNP levels at admission, divided by the cut-off point for the different candidate cut points tested.

As NT-proBNP cut-off points vary depending on the clinical setting and patient characteristics, we analyzed the effect size of P-IABP compared to R-IABP on the primary endpoint for different candidate cut-off points. In the subgroup with NT-proBNP levels ≥ 10,000 pg/mL, the effect size yielded a Cohen‘s d of 5.01 (95% CI 2.48–7.64), demonstrating a strong beneficial effect of P-IABP. For patients with NT-proBNP levels below this cut-off point, Cohen’s d was −0.05 (95% CI −0.89 to − 0.79), not even showing a small effect. For the cut-off point of 5000 pg/mL, a strong but less pronounced beneficial effect of P-IABP was observed in patients with levels equal to or above the cut point (Cohen’s d = 1.17, 95% CI −0.05–2.39), while there was a small beneficial effect of R-IABP in patients with lower NT-proBNP levels (Cohen’s d = −0.37, 95% CI −1.46–0.72). The effect size for patients exceeding the cut-off point of 900 pg/mL was even lower and only moderate (Cohen’s d = 0.70, 95% CI −0.09–1.49). However, in the subgroup with NT-proBNP levels below this cut-off, there was a strong beneficial effect of R-IABP (Cohen’s d = −0.90, 95% CI −8.97 to −7.19). The effect sizes of P-IABP compared to R-IABP for the aforementioned subgroups for all tested cut-offs are shown in Figure 4.

### 3.8. Clinical Implications of Peri-Interventional Myocardial Damage Assessed by Mortality

A total of seven patients (11.9%) died within 14 days, with no further fatalities up to the 30-day follow-up. Of these, five patients received P-IABP and two patients were treated with R-IABP, corresponding to mortality rates of 11.4% and 13.3% (*p* = 0.842). Patients who died within 14 and 30 days had a descriptively higher peri-interventional increase in hsTnT levels than patients alive at this time point, although this was not statistically significant (each 2472.2 ± 4208.93 vs.1346.1 ± 2954.26 ng/mL, *p* = 0.447). Cox regression analysis showed that a high peri-interventional hsTnT increase was significantly associated with a shorter survival time (*p* = 0.046).

The predictors of low RRsyst (*p* = 0.004, r = 0.349) respectively hypotension (*p* = 0.015, r = 0.365), as well as high NT-proBNP levels (*p* = 0.013, r = 0.404), were significantly correlated with 30-day mortality.

The rates of mortality and Major Adverse Cardiovascular Events (MACE), defined as cardiovascular emergency hospitalization or death, are shown in Table 6.

## 4. Discussion

This study investigated criteria to identify patients undergoing high-risk PCI who would benefit from early pre-interventional P-IABP implantation compared to later peri-procedural R-IABP implantation. Overall, P-IABP implantation was associated with lower peri-interventional myocardial injury, as indicated by hsTnT increase, which was associated with worse survival. Specifically, R-IABP patients with ST-segment elevation (r = 0.631), low systolic blood pressure (r = 0.893), and high NT-proBNP levels (r = 0.953) had a high peri-interventional hsTnT increase. Importantly, in patients with low systolic blood pressure and high NT-proBNP levels, this association was significantly weaker in the P-IABP group. Specifically, patients without hypertension but with low normal blood pressure values and patients with NT-proBNP levels ≥ 900 pg/mL benefited from P-IABP. Consequently, these criteria can help set the indications for P-IABP.

### 4.1. Clinical Implications of Peri-Interventional hsTnT Increase

To further investigate the clinical relevance of the endpoint of peri-interventional hsTnT increase, we examined its correlation with the clinical outcome of death. While overall mortality rates between the groups were comparable, showing no general clinical benefit of P-IABP compared to R-IABP, the association between peri-interventional hsTnT increase and mortality rates indicates that patients experiencing lower hsTnT increases also experience improved survival. This association between peri-interventional hsTnT increase and mortality can be explained by the correlation between this biomarker and infarct size [22]. These data confirm the suitability of peri-interventional hsTnT increase as an endpoint for assessing the outcomes of PCI.

### 4.2. P-IABP Is Beneficial Overall

Despite the subjective nature of determining IABP indications, our study demonstrates that P-IABP implantation is beneficial overall in patients undergoing high-risk PCI. With regard to the timing of IABP implantation, prophylactic pre-interventional insertion of the device significantly reduced myocardial injury, as evidenced by lower peri-interventional hsTnT increases compared to a later peri-interventional rescue implantation. While the comparable mortality rates between the P- and R-IABP group indicate no general benefit of P-IABP in our cohort, the descriptively higher peri-interventional hsTnT increase in patients who died and, most importantly, the significant association between peri-interventional hsTnT increase and survival time, show that peri-interventional ischemia is associated with mortality. Consequently, our results suggest that reducing the peri-interventional increase in infarct size, as observed with P-IABP implantation in at-risk patients, may improve their survival. This is particularly important because the identified risk factors of increased ischemia, low RRsyst, and high NT-proBNP levels were associated with higher mortality rates, emphasizing the clinical relevance of identifying a promising treatment strategy for these patients. This result aligns with prior findings showing improved outcomes with P-IABP. Even in the 1970s, a study on patients without cardiogenic shock undergoing cardiac surgery who received IABP insertion due to inability to wean showed that elective early IABP implantation improves survival [23]. Studies on patients undergoing high-risk PCI reported that patients receiving P-IABP had higher procedural success rates, lower mortality, and fewer major complications both during the in-hospital stay and at six months compared to R-IABP patients [24]. P-IABP implantation was found to be the only independent predictor of survival at six months [24] and a predictor of freedom from catheterization laboratory events [25]. In conclusion, these data support the notion that P-IABP represents a beneficial strategy and should therefore be considered in the context of high-risk PCI.

### 4.3. Need for Pre-Selection

However, it is important to note that the association between prophylactic insertion and peri-interventional myocardial injury was only moderate, with a correlation strength of r = 0.380. This raises the question of whether rescue or bailout implantation may be a viable alternative, as this approach would allow for more efficient patient selection, with IABP reserved for those who really need it. Accordingly, we observed fewer R-IABP than P-IABP implantations, suggesting a reduction in overall IABP use under the rescue strategy. In this context, balancing the risks and benefits of invasive procedures is crucial. IABP insertion carries potential complications, such as bleeding, ischemia, thrombosis, and infection [26]. However, it is noteworthy that elective use has shown low morbidity [23], which may be due to the controlled setting, whereas R-IABP was performed due to unexpected hemodynamic worsening. Accordingly, the incidence of vascular complications has been reported to be low overall and non-inferior in P-IABP, with even higher rates of major bleeding in R-IABP patients [24]. Additionally, another risk is that IABP may reduce coronary flow in some patients with severe coronary artery disease [27], probably impairing autoregulatory function, as indicated by increased aortic diastolic pressure through increased flow to areas supplied by collateral vessels or critical stenoses [28]. In our study, peri-interventional complication rates were similar for patients undergoing P- and R-IABP implantation, demonstrating that a rescue strategy does not impair patient safety with regard to device implantation complications. Given the demonstrated benefits and risks of each strategy, the overall reduced myocardial injury with P-IABP, the low but present risk of complications associated with IABP implantation [26], and the improved patient selection with R-IABP, the urgent need for evidence-based criteria to guide patient selection for P-IABP becomes evident.

### 4.4. Current Selection Criteria Are Weak

The current literature, as exemplified by the CRISP-AMI study, indicates that not all patients benefit from IABP therapy. However, the criteria for identifying patients who would benefit from this treatment remain elusive [29]. To date, the criteria for the indication of P-IABP remain a subject of debate, and its use in high-risk PCI varies across hospitals [30]. Cardiac physiology is the primary consideration when deciding whether to perform protected PCI [31,32,33]. In our study, the decision for P-IABP implantation prior to PCI was also made at the discretion of the interventionalists. According to the definitions and the Society for Cardiovascular Angiography and Interventions (SCAI)/American College of Cardiology (ACC)/Heart Failure Society of America (HFSA)/Society of Thoracic Surgeons (STS) 2015 expert consensus statement [14,15], prophylactic hemodynamic support should be considered in cases of complex PCI and low patient reserve, particularly in instances of heart failure with reduced LVEF. In patients fulfilling only one of these criteria, the use of IABP or Impella is recommended as a backup [14,15]. Notably, the assessment of the expected complexity of the procedure, and thus the necessity for IABP support, depends on the interventionalist’s experience. The limitations of this assessment are demonstrated by the results of our study, which showed comparable complexity of the coronary lesion as measured by the Syntax score in both the P- and R-IABP group and thus did not reflect the subjective decision. Efforts to assist clinicians in deciding on the indication of P-IABP implantation have led to an algorithm aimed at predicting intraprocedural decompensation and the potential benefit of up-front protected PCI. In addition to LVEF, this algorithm incorporates other indicators of cardiac function, including the cardiac index, pulmonary oxygen saturation, and severe mitral regurgitation. This schema also considers complexity, as indicated by the Syntax score, presentation as acute coronary syndrome, planned multivessel intervention, and conditions reducing patient reserve, such as low systolic blood pressure, decompensated clinical state, at-risk vascular injury, severe anemia, and likely prolonged ischemia [14,33]. Elevated left ventricular end-diastolic pressure (LVEDP) is also considered a risk factor, as it is associated with ischemic hypotension resulting from reduced coronary perfusion [34]. However, right heart catheterization (RHC) is required to assess filling pressures, cardiac index, and power, which is clearly not feasible in an emergency setting, as in the majority of cases in our study [14]. The aforementioned criteria are supported by a study on patients with acute myocardial infarction, which found that IABP implantation before intervention reduces catheterization laboratory events in patients with cardiogenic shock, congestive heart failure, low ejection fraction, and all high-risk patients combined [25]. Similarly, another investigation on P-IABP support during cardiac surgery found that, particularly patients with severe preoperative left ventricular dysfunction or a combination of moderate dysfunction with coronary or valvular pathology, exhibited enhanced survival rates through this measure [23]. Consequently, we considered these parameters in our analysis. Notably, in our study, LVEF was not significantly different between the P-IABP and R-IABP group, suggesting that this criterion did not contribute to the decision. The lack of objective criteria for P-IABP use in clinical practice is further emphasized by the similarity of the remaining patients’ demographics, comorbidities, and parameters of cardiac function in our study. These data highlight the need for a reliable strategy to identify at-risk patients who will benefit from P-IABP based on their baseline characteristics and conditions.

### 4.5. Absence of Hypertension and Heart Failure Indicate Benefit of P-IABP

Notably, we found that the routinely assessed criteria of blood pressure and NT-proBNP as an indicator of heart failure correlated with the benefit from pre-interventional, early, P-IABP insertion and can therefore serve as key criteria for indicating P-IABP in high-risk PCI.

Specifically, our results show that patients with low systolic blood pressure benefit from early pre-interventional IABP (P-IABP) implantation. While there was a strong correlation between low systolic blood pressure and high peri-interventional myocardial injury as measured by hsTnT increase (r = 0.893) in R-IABP patients, this association was lower and not significant in patients receiving P-IABP, and patients without hypertensive blood pressure values at admission had significantly lower peri-interventional hsTnT increases when treated with P-IABP. This benefit of early insertion can be explained by the loss of IABP efficiency once hemodynamic insufficiency progresses to cardiogenic shock [13]. As already known, the effectiveness of IAPB declines when systolic blood pressure falls below 60 mmHg or heart rate exceeds 140 beats per minute (bpm) due to technical and cardiac limitations [35,36]. This is in line with the diagnostic algorithm proposed by Kerney et al., which lists low systolic blood pressure as a criterion for selecting patients for protected PCI [14,33]. These data indicate that low blood pressure should be a criterion for early P-IABP implantation.

Beyond this clinical criterion of low blood pressure, our study found that high levels of the laboratory biomarker NT-proBNP may indicate a benefit of pre-interventional P-IABP implantation. High NT-proBNP levels correlated with greater peri-interventional myocardial injury, as indicated by the strong correlation with peri-interventional hsTnT increases (r = 0.953) in the R-IABP group, whereas this association was lower and not significant in the P-IABP group. In particular, in patients with NT-proBNP levels indicating acute, (very) severe heart failure of ≥10,000 and ≥5000 pg/mL [19,20], a strong beneficial effect of P-IABP was observed, which declined for lower levels to only a moderate effect for ≥900 pg/mL. Interestingly, among patients with NT-proBNP levels below the age-specific cut-off point for heart failure of 900 pg/mL [18], there was a strong beneficial effect of R-IABP on this endpoint. Considering these results, the threshold at which the benefits of P-IABP come into effect appears to correspond to the age-specific NT-proBNP cut-off point for heart failure. As the gold standard for diagnosing and predicting the prognosis of heart failure [37], NT-proBNP indicates cardiac impairment, and thus encompasses several criteria enumerated in the aforementioned decision algorithm for the indication of P-IABP use proposed by Kerney [14,33]. This finding underscores the utmost importance of preserving cardiac function by initiating cardiac support prior to significant deterioration, which aligns with the declining effect of IABP support in cardiogenic shock discussed above [9,35,36]. The increasing size of the beneficial effect of P-IABP and diminishing effect of R-IABP as NT-proBNP levels rise toward levels indicative of acute severe heart failure [20,21] emphasizes the urgent need for early, prophylactic implantation of IABP in affected patients. Therefore, we can demonstrate that P-IABP constitutes a highly effective strategy to prevent the well-documented adverse prognostic and clinical implications associated with exceeding this threshold for acute, very severe heart failure [18,19,20]. In contrast, the beneficial effect of R-IABP in patients with NT-proBNP levels indicating the absence of heart failure may be explained by an improved pre-selection through this strategy, as previously discussed, and the potential complication risks associated with IABP implantation [26]. In view of these data, NT-proBNP could assist clinicians in making objective and reproducible decisions about pre-interventional P-IABP implantation in high-risk PCI, with the aforementioned thresholds serving as guides for ruling in or ruling out.

While patients inheriting the aforementioned criteria benefited from P-IABP, the criterion ST-segment elevation correlated in both the R-IABP and P-IABP group, with peri-interventional myocardial injury as indicated by peri-interventional hsTnT increase, with no significant difference in the strength of the correlation. This finding suggests that ST-segment elevation is associated with this endpoint, regardless of the timing of IABP insertion. This may explain why we did not observe the benefit of P-IABP implantation in these patients, which may be due to the high degree of myocardial injury characteristic of this condition. However, these patients are at an increased risk for R-IABP, as ST-segment elevation was more common in this group in our study. Importantly, Brodie et al. found that in patients with acute myocardial infarction complicated by heart failure, pre-interventional IABP led to better outcomes than post-interventional insertion [25], indicating that further delay may be critical. As we did not observe a benefit through pre-interventional P-IABP implantation compared to peri-interventional rescue implantation in patients with ST-segment elevation, according to our data, IABP implantation should not be performed at the cost of delaying revascularization procedures in these patients. However, the effects of standard IABP implantation immediately thereafter must be evaluated.

While the current guidelines recommend parameters such as complex coronary situation and low LVEF for the decision of P-IABP [14,15,34], the present analysis does not provide evidence that they independently predict a benefit of early prophylactic, compared to later R-IABP implantation. As we did not observe an association between these parameters and peri-interventional hsTnT increase in the R-IABP group, there is no evidence that these patients are at particular risk of circulatory deterioration. However, these conditions are likely to affect the identified predictors of blood pressure and NT-proBNP levels and may thus be indirectly associated with the benefit of P-IABP.

Overall, our data show that patients with low cardiac or hemodynamic reserve benefit from early prophylactic IABP implantation. This need to prevent critical deterioration can be explained by the drastic consequences of cardiogenic shock and increasing mortality as the shock progresses [10]. This demonstrates the urgent need to assist clinicians in making decisions regarding P-IABP implantation.

### 4.6. Limitations

The results of our study must be interpreted with several limitations. As this was a retrospective, observational, single-center study, it had several limitations inherent to this design. Due to this design, the sample size was small and varied between groups. However, all consecutive patients during the defined time period were included without patient exclusion or pre-selection, aiming to reduce selection bias as much as possible. Although the strength of the observed effect supports the robustness of the reported association within the current sample, the small sample size may limit generalizability to other populations. In addition, the small sample size may result in increased variability, leading to effect size inflation and smaller effects being underreported, as they may not have reached statistical significance. However, given the strong and significant effect of P-IABP on infarct size in at-risk patients and its relevance to clinical outcomes, we consider further patient enrollment neither necessary nor ethically justifiable. Consequently, we considered it essential to stop the trial at this point to ensure that at-risk patients have access to the P-IABP. Additionally, due to the explorative nature of the study, all results must be interpreted as generating hypotheses. Moreover, the higher prevalence of ST-segment elevation in the R-IABP group could impact the endpoint of peri-interventional hsTnT increase. We addressed this using partial correlation analyses, controlling for ST-segment elevation. We also acknowledge that a subgroup analysis limited to non-STEMI patients could provide additional insight; however, this was not statistically feasible due to the small remaining sample size. Furthermore, the decision regarding P-IABP implantation may be subject to debate. The criterion ‘complexity of PCI’ was evaluated by the interventionalist in accordance with the guideline recommendations; however, although all interventions were conducted at our center, this assessment may vary depending on the interventionalist’s experience.

## 5. Conclusions

Given the overall beneficial effect of early, pre-procedure, (P)rophylactic IABP implantation compared to later, peri-interventional, (R)escue IABP implantation and the risks associated with this invasive measure, this study sought to investigate the criteria for identifying patients at risk of peri-interventional myocardial injury during high-risk PCI while benefiting from P-IABP. Our results confirm the benefit of P-IABP in high-risk PCI overall, as this measure was associated with less peri-interventional myocardial injury, as measured by hsTnT increase, which was clinically associated with worse survival. Importantly, we found that patients with low systolic blood pressure, in particular those without hypertension, and high NT-proBNP levels indicative of heart failure, benefit from P-IABP, as early pre-interventional implantation reduces the risk of high peri-interventional myocardial injury seen with R-IABP. However, the strong beneficial effect of R-IABP in patients with NT-proBNP levels indicating the absence of heart failure highlights the importance of carefully determining this indication. Consequently, we agree with hemodynamic and cardiac conditions as criteria for P-IABP implantation prior to high-risk PCI but plead for the establishment of clear, objective criteria for assessment.

## Figures and Tables

**Table 1:** Baseline characteristics of patients in the P-IABP and R-IABP group.

	P-IABP	R-IABP	*p*-Value
	*n* = 44	*n* = 15	
**Demographics**			
Age (years)	74.02 ± 9.56	70.93 ± 12.57	0.33
Male sex *n*, (%)	30 (68.2)	12 (80)	0.38
Emergency referral *n*, (%)	34 (77.3)	15 (100)	**0.04**
Syntax score	31.65 ± 8.94	31.33 ± 8.95	0.91
History of coronary revascularization total *n*, (%)	21 (47.7)	8 (53.3)	0.71
History of stent implantation *n*, (%)	19 (43.2)	6 (40.0)	0.83
History of bypass surgery *n*, (%)	2 (4.5)	2 (13.3)	0.25
**Clinical Presentation**			
Heart rate pre-intervention (bpm)	83.15 ± 20.15	83.71 ± 17.70	0.93
RRsyst pre-intervention (mmHg)	131.97 ± 22.80	125.55 ± 22.48	0.42
RRdiast pre-intervention (mmHg)	71.28 ± 11.46	70.82 ± 12.24	0.91
**Imaging**			
LVEF via laevocardiography (grade)	3.44 ± 1.01	3.73 ± 0.46	0.43
normal (1) *n*, (%)	5 (11.4)	0 (0)	
mildly impaired (2) *n*, (%)	1 (2.3)	0 (0)	
moderately impaired (3) *n*, (%)	7 (15.9)	4 (26.7)	
severely impaired (4) *n*, (%)	31 (70.5)	11 (73.3)	
Severe aortic valve stenosis *n*, (%)	3 (7.9)	1 (8.3)	0.96
Severe mitral regurgitation *n*, (%)	6 (15.8)	1 (8.3)	0.52
sPAP (mmHg)	52.61 ± 14.87	57.80 ± 12.32	0.48
**ECG**			
Sinus rhythm *n*, (%)	40 (90.9)	15 (100)	0.23
Atrial tachycardia (Atrial fibrillation/Atrial flutter) *n*, (%)	4 (9.1)	0 (0.0)	0.23
Left bundle branch block *n*, (%)	9 (21.4)	3 (20.0)	0.96
Right bundle branch block *n*, (%)	5 (12.2)	4 (30.8)	0.12
ST-segment elevation *n*, (%)	10 (22.7)	9 (60.0)	**0.01**
ST-segment depression *n*, (%)	13 (31.0)	8 (53.3)	0.12
T-wave inversion *n*, (%)	40 (90.9)	12 (80.0)	0.26
**Lab**			
hsTnT (ng/L)	738.76 ± 1339.61	423.0 ± 787.22	0.46
NT-proBNP (pg/mL)	11,836 ± 11,279	10,443 ± 11,688	0.75
GFR (ml/min)	56.5 ± 25.07	53 ± 22.52	0.67
Creatinine (µmol/L)	126.97 ± 72.75	127.62 ± 60.79	0.98
CK (U/L)	431.81 ± 849.13	258.67 ± 404.04	0.56
**Coronary State**			
Left Main Stenosis *n*, (%)	31 (73.8)	7 (46.7)	0.08
Ostial RCA stenosis *n*, (%)	21 (51.2)	7 (46.7)	0.77
**PCI Procedure**			
Staged PCI *n*, (%)	8 (18.2)	0 (0)	**0.003**
Indication for Coronary Angiography			**0.002**
Acute Coronary Syndrome *n*, (%)	30 (68.8)	14 (93.3)	
Acute Heart Failure *n*, (%)	5 (11.4)	1 (6.7)	
Chronic Heart Failure *n*, (%)	8 (20.5)	0(0)	

P-IABP, Prophylactic-Intra-Aortic Balloon Pump; R-IABP, Rescue-Intra-Aortic Balloon Pump; bpm, beats per minute; RRsyst, systolic blood pressure (measured according to Riva Rocci); RRdiast, diastolic blood pressure (measured according to Riva Rocci); LVEF, left ventricular systolic function; sPAP; systolic pulmonary artery pressure; hsTnT, high-sensitivity Troponin T; NT-proBNP, N-terminal pro-brain natriuretic peptide; GFR, Glomerular Filtration Rate (estimated by Cockroft Gault Formula); CK, creatine kinase; RCA, Right coronary artery; PCI, Percutaneous Coronary Intervention; significant differences are presented in bold.

**Table 2:** Procedural data of patients in the P-IABP and R-IABP group.

	P-IABP	R-IABP	*p*-Value
	*n* = 44	*n* = 15	
**Procedural Parameters**			
Intervention length (hours)	1.54 ± 0.65	1.49 ± 0.60	0.78
Periprocedural CPR *n*, (%)	1 (2.3)	0 (0.0)	0.55
Periprocedural defibrillation *n*, (%)	3 (7.0)	2 (13.3)	0.45
Periprocedural cardiogenic shock *n*, (%)	1 (2.4)	0 (0.0)	0.55
**Primary vessel of intervention**	(*n* = 41)	(*n* = 13)	
LAD *n*, (%)	10 (24.4)	5 (38.4)	0.43
RCX *n*, (%)	3 (7.3)	2 (15.4)	
RCA *n*, (%)	5 (12.2)	2 (15.4)	
Left main *n*, (%)	23 (56.1)	4 (30.8)	
Initial TIMI flow (grade)	1.37 ± 0.75	1.28 ± 0.88	0.66
Number of vessels remaining stenosed after index procedure	1.22 ± 0.99	0.93 ± 0.95	0.32
Number of stents placed	3.02 ± 1.76	2.47 ± 1.45	0.26
**Access Site Complication Rates**			
IABP access site femoral *n* (%)	3 (6.8)	0 (0)	0.304
Coronary Angiography/PCI access site, radial *n* (%)	1 (2.3)	0 (0)	0.564

P-IABP, Prophylactic-Intra-Aortic Balloon Pump; R-IABP, Rescue-Intra-Aortic Balloon Pump; CPR, cardiopulmonary resuscitation; LAD, Left anterior descending artery; RCX, Ramus circumflexus; RCA, Right coronary artery; TIMI, Thrombolysis in Myocardial Infarction; PCI, Percutaneous Coronary Intervention.

**Table 3:** HsTnT levels before and after the intervention, along with the pre-interventional increase in patients in the P-IABP and R-IABP groups.

	P-IABP	R-IABP	*p*-Value
	*n* = 44	*n* = 15	
hsTnT before PCI (ng/L)	738.76 ± 1339.61	423.0 ± 787.22	0.46
Time until next hsTnT measurement (hours)	3.68 ± 6.15	3.45 ± 4.63	0.89
hsTnT immediately after PCI (ng/L) *	1411.81 ± 2451.34	4557.15 ± 4306.72	**0.001**
Peri-interventional hsTnT increase (ng/L) *	767.85 ± 2199.05	3538.91 ± 4421.74	0.069

hsTnT, high-sensitivity Troponin T; P-IABP, Prophylactic-Intra-Aortic Balloon Pump; R-IABP, Rescue-Intra-Aortic Balloon Pump; hsTnT, PCI, Percutaneous coronary intervention; significant differences are presented in bold. * Levels above 10,000 ng/L could not be determined and were counted as 10,000 ng/L.

**Table 4:** Pearson correlation analysis and Spearman’s rank correlation for baseline parameters with peri-interventional hsTnT Increase in the R-IABP Group.

	*p*-Value	R-Value	95%-CI
Male sex	0.945	−0.024	−0.615, 0.585
Syntax score	0.423	−0.269	−0.748, 0.394
History of cardiovascular revascularization	0.377	−0.303	−0.764, 0.363
History of coronary artery bypass graft	0.820	−0.078	−0.547, 0.648
History of coronary stenting	0.571	−0.412	−0.916, 0.600
**RRsyst pre-intervention (mmHg)**	**0.007**	**−0.893**	**−0.984, −0.427**
RRdiast pre-intervention (mmHg)	0.828	−0.102	−0.794, 0.705
LVEF (grade)	0.899	−0.044	−0.627, 0.571
Severe aortic valve stenosis	0.540	0.256	−0.814, 0.547
Severe mitral regurgitation	0.540	−0.256	−0.814, 0.547
sPAP (mmHg)	0.618	0.305	−0.790, 0.936
Left bundle branch block	0.976	0.010	−0.593, 0.606
Right bundle branch block	0.641	0.159	−0.488, 0.693
**ST-segment elevation**	**0.037**	**0.631**	**0.050, 0.893**
ST-segment depression	0.819	−0.078	−0.648, 0.547
T-wave inversion	0.310	0.104	−0.529, 0.663
hsTnT at admission (ng/L)	0.660	−0.150	−0.688, 0.494
**NT-proBNP (pg/mL)**	**<0.001**	**0.953**	**0.708, 0.993**
Creatinine (µmol/L)	0.405	0.279	−0.385, 0.753
CK (U/L)	0.499	−0.310	−0.862, 0.578
Left main stenosis	0.381	−0.293	−0.760, 0.372
Proximal RCA stenosis	0.625	−0.166	−0.697, 0.482

CI, Confidence Interval; hsTnT, high-sensitivity Troponin T; R-IABP, Rescue-Intra-Aortic Balloon Pump; RCA, Right coronary artery; LVEF, left ventricular systolic function; sPAP; systolic pulmonary artery pressure; RRsyst, systolic blood pressure (measured according to Riva Rocci); RRdiast, diastolic blood pressure (measured according to Riva Rocci); NT-proBNP, N-terminal pro-brain natriuretic peptide; CK, creatine kinase; significant differences are presented in bold.

**Table 5:** Pearson correlation analysis and Spearman’s rank correlation for baseline parameters significantly correlated with peri-interventional hsTnT increase in the P-IABP group, with peri-interventional hsTnT Increase in the R-IABP Group.

	*p*-Value	r-Value	95%-CI
RRsyst pre-intervention (mmHg)	0.856	−0.036	−0.404, 0.342
ST-segment elevation	0.074	0.310	−0.031, 0.584
NT-proBNP (pg/mL)	0.762	0.051	−0.336, 0.423

CI, Confidence Interval; hsTnT, high-sensitivity Troponin T; P-IABP, Prophylactic-Intra-Aortic Balloon Pump; R-IABP, Rescue-Intra-Aortic Balloon Pump; RRsyst, systolic blood pressure (measured according to Riva Rocci); NT-proBNP, N-terminal pro-brain natriuretic peptide.

**Table 6:** Post-interventional event rates in patients in the P-IABP and R-IABP group.

	P-IABP	R-IABP	*p*-Value
	*n* = 44	*n* = 15	
14-day Mortality (%)	5 (11.4)	2 (13.3)	0.447
30-day Mortality (%)	5 (11.4)	2 (13.3)	0.447
180-day Mortality (%)	8 (18.2)	3 (20)	0.879
365-day Mortality (%)	10 (22.7)	3 (20)	0.829
365-day MACE (%)	15 (34.1)	5 (33.3)	0.959

MACE, Major Adverse Cardiovascular Events, defined as cardiovascular emergency hospitalization or death; P-IABP, Prophylactic-Intra-Aortic Balloon Pump; R-IABP, Rescue-Intra-Aortic Balloon Pump.

## Data Availability

The datasets presented in this article are not readily available because of technical restrictions. Requests for access to the datasets should be directed to the corresponding authors.

## Abbreviations

ACC	American College of Cardiology
Bpm	beats per minute
CK	Creatine Kinase
CRISP-AMI	Counterpulsation to Reduce Infarct Size Pre-PCI Acute Myocardial Infarction trial
CSI	Cardiogenic Shock Initiative
CI	Confidence Interval
d	Cohen’s d (effect size measure)
ECG	Electrocardiogram
ECMO	Extracorporeal Membrane Oxygenation
ECLIA	ElectroChemiLumineszenz ImmunoAssay
ESC	European Society of Cardiology
GFR	Glomerular Filtration Rate
HFSA	Heart Failure Society of America
hsTnT	High-sensitivity Troponin T
IABP	Intra-Aortic Balloon Pump
IQR	Interquartile Range
IZI	Fisher’s Z-transformed difference in correlation coefficients
LAD	Left Anterior Descending artery
LVSF/LVEF	Left Ventricular Systolic Function/Left Ventricular Ejection Fraction
LVAD	Left Ventricular Assist Device
LVEDP	Left Ventricular End-Diastolic Pressure
MACE	Major Adverse Cardiovascular Events
NT-proBNP	N-terminal pro-Brain Natriuretic Peptide
PCI	Percutaneous Coronary Intervention
P-IABP	Prophylactic-Intra-Aortic Balloon Pump
RCA	Right Coronary Artery
RHC	Right Heart Catheterization
R-IABP	Rescue-Intra-Aortic Balloon Pump
RRdiast	Diastolic Blood Pressure (Riva Rocci)
RRsyst	Systolic Blood Pressure (Riva Rocci)
sPAP	systolic Pulmonary Artery Pressure
SCAI	Society for Cardiovascular Angiography and Interventions
SPSS	Statistical Package for the Social Sciences
STS	Society of Thoracic Surgeons
TIMI	Thrombolysis in Myocardial Infarction

## References

[1] Krishna M., Zacharowski K. (2009). Principles of intra-aortic balloon pump counterpulsation. Contin. Educ. Anaesth. Crit. Care Pain.

[2] Khan T.M., Siddiqui A.H. (2024). Intra-Aortic Balloon Pump. StatPearls.

[3] Miller P.E., Bromfield S.G., Ma Q., Crawford G., Whitney J., DeVries A., Desai N.R. (2022). Clinical Outcomes and Cost Associated with an Intravascular Microaxial Left Ventricular Assist Device vs. Intra-aortic Balloon Pump in Patients Presenting with Acute Myocardial Infarction Complicated by Cardiogenic Shock. JAMA Intern. Med..

[4] Dhruva S.S., Ross J.S., Mortazavi B.J., Hurley N.C., Krumholz H.M., Curtis J.P., Berkowitz A., Masoudi F.A., Messenger J.C., Parzynski C.S. (2020). Association of Use of an Intravascular Microaxial Left Ventricular Assist Device vs. Intra-aortic Balloon Pump with In-Hospital Mortality and Major Bleeding Among Patients with Acute Myocardial Infarction Complicated by Cardiogenic Shock. JAMA.

[5] Javaid A.I., Michalek J.E., Gruslova A.B., Hoskins S.A., Ahsan C.H., Feldman M.D. (2024). Mechanical circulatory support versus vasopressors alone in patients with acute myocardial infarction and cardiogenic shock undergoing percutaneous coronary intervention. Catheter. Cardiovasc. Interv..

[6] O’Neill W.W., Kleiman N.S., Moses J., Henriques J.P.S., Dixon S., Massaro J., Palacios I., Maini B., Mulukutla S., Dzavik V. (2012). A prospective, randomized clinical trial of hemodynamic support with Impella 2.5 versus intra-aortic balloon pump in patients undergoing high-risk percutaneous coronary intervention: The PROTECT II study. Circulation.

[7] Alushi B., Douedari A., Froehlig G., Knie W., Wurster T.H., Leistner D.M., Staehli B.-E., Mochmann H.-C., Pieske B., Landmesser U. (2019). Impella versus IABP in acute myocardial infarction complicated by cardiogenic shock. Open Heart.

[8] Parissis H., Graham V., Lampridis S., Lau M., Hooks G., Mhandu P.C. (2016). IABP: History-evolution-pathophysiology-indications: What we need to know. J. Cardiothorac. Surg..

[9] Powell W.J., Daggett W.M., Magro A.E., Bianco J.A., Buckley M.J., Sanders C.A., Kantrowitz A.R., Austen W.G. (1970). Effects of intra-aortic balloon counterpulsation on cardiac performance, oxygen consumption, and coronary blood flow in dogs. Circ. Res..

[10] Hill K.L., Rustin M.A., Asche M.A., Bennett C.E., Patel P.C., Jentzer J.C. (2023). Cardiogenic Shock Classification and Associated Mortality Risk. Mayo Clin. Proc..

[11] Basir M.B., Schreiber T.L., Grines C.L., Dixon S.R., Moses J.W., Maini B.S., Khandelwal A.K., Ohman E.M., O’Neill W.W. (2017). Effect of Early Initiation of Mechanical Circulatory Support on Survival in Cardiogenic Shock. Am. J. Cardiol..

[12] Archilletti F., Giuliani L., Dangas G.D., Ricci F., Benedetto U., Radico F., Gallina S., Rossi S., Maddestra N., Zimarino M. (2022). Timing of mechanical circulatory support during primary angioplasty in acute myocardial infarction and cardiogenic shock: Systematic review and meta-analysis. Catheter. Cardiovasc. Interv..

[13] Perera D., Stables R., Thomas M., Booth J., Pitt M., Blackman D., de Belder A., Redwood S. (2010). BCIS-1 InvestigatorsBCIS-1 Investigators. Elective intra-aortic balloon counterpulsation during high-risk percutaneous coronary intervention: A randomized controlled trial. JAMA.

[14] Kearney K.E., McCabe J.M., Riley R.F. (2019). Hemodynamic Support for High-Risk PCI. Card. Interv. Today.

[15] Rihal S.C., Naidu S.S., Givertz M.M., Szeto W.Y., Burke J.A., Kapur N.K., Kern M., Garratt K.N., Goldstein J.A., Dimas V. (2015). 2015 SCAI/ACC/HFSA/STS clinical expert consensus statement on the use of percutaneous mechanical circulatory support devices in cardiovascular care: Endorsed by the American Heart Association, the Cardiological Society of India, and Sociedad Latino Americana de Cardiologia Intervencion; affirmation of value by the Canadian Association of Interventional Cardiology-Association Canadienne de Cardiologie d’intervention. J. Am. Coll. Cardiol..

[16] Thiele H., Zeymer U., Neumann F.J., Ferenc M., Olbrich H.G., Hausleiter J., Richardt G., Hennersdorf M., Empen K., Fuernau G. (2012). Intraaortic Balloon Support for Myocardial Infarction with Cardiogenic Shock. N. Engl. J. Med..

[17] Ponikowski P., Voors A.A., Anker S.D., Bueno H., Cleland J.G.F., Coats A.J.S., Falk V., González-Juanatey J.R., Harjola V.-P., Jankowska E.A. (2016). ESCScientific Document Group 2016 ESCGuidelines for the diagnosis treatment of acute chronic heart failure: The Task Force for the diagnosis treatment of acute chronic heart failure of the European Society of Cardiology (ESC)Developed with the special contribution of the Heart Failure Association (HFA) of the, ESC. Eur. Heart J..

[18] Januzzi J.L., van Kimmenade R., Lainchbury J., Bayes-Genis A., Ordonez-Llanos J., Santalo-Bel M., Pinto Y.M., Richards M. (2006). NT-proBNP testing for diagnosis and short-term prognosis in acute destabilized heart failure: An international pooled analysis of 1256 patients: The International Collaborative of NT-proBNP Study. Eur. Heart J..

[19] Trang T.T.H. Value of NT-proBNP Test in Diagnosis, Monitoring, Prognosis and Screening of Heart Failure. Value of NT-proBNP Test in Diagnosis, Monitoring, Prognosis and Screening of Heart Failure. https://www.vinmec.com.

[20] Bózsik B., Nagy E., Somlói M., Tomcsányi J. (2017). Extrém magas B típusú natriureticus prohormon prognosztikai szerepe a szívelégtelenség miatt kezelt betegek kórházi halálozására [The prognostic role of extremely high levels of the B-type natriuretic prohormone with regard to the in-hospital mortality of patients hospitalized for heart failure]. Orv. Hetil..

[21] Whelton P.K., Carey R.M., Aronow W.S., Casey D.E., Collins K.J., Himmelfarb C.D., DePalma S.M., Gidding S., Jamerson K.A., Jones D.W. (2018). 2017 ACC/AHA/AAPA/ABC/ACPM/AGS/APhA/ASH/ASPC/NMA/PCNA Guideline for the Prevention, Detection, Evaluation, and Management of High Blood Pressure in Adults: A Report of the American College of Cardiology/American Heart Association Task Force on Clinical Practice Guidelines. Hypertension.

[22] Giannitsis E., Steen H., Kurz K., Ivandic B., Simon A.C., Futterer S., Schild C., Isfort P., Jaffe A.S., Katus H.A. (2008). Cardiac Magnetic Resonance Imaging Study for Quantification of Infarct Size Comparing Directly Serial Versus Single Time-Point Measurements of Cardiac Troponin T. J. Am. Coll. Cardiol..

[23] Bolooki H., Williams W., Thurer R.J., Vargas A., Kaiser G.A., Mack F., Ghahramani A.R. (1976). Clinical and hemodynamic criteria for use of the intra-aortic balloon pump in patients requiring cardiac surgery. J. Thorac. Cardiovasc. Surg..

[24] Mishra S., Chu W.W., Torguson R., Wolfram R., Deible R., Suddath W.O., Pichard A.D., Satler L.F., Kent K.M., Waksman R. (2006). Role of prophylactic intra-aortic balloon pump in high-risk patients undergoing percutaneous coronary intervention. Am. J. Cardiol..

[25] Brodie B.R., Stuckey T.D., Hansen C., Muncy D. (1999). Intra-aortic balloon counterpulsation before primary percutaneous transluminal coronary angioplasty reduces catheterization laboratory events in high-risk patients with acute myocardial infarction. Am. J. Cardiol..

[26] Parissis H., Soo A., Al-Alao B. (2011). Intra aortic balloon pump: Literature review of risk factors related to complications of the intraaortic balloon pump. J. Cardiothorac. Surg..

[27] Port S.C., Patel S., Schmidt D.H. (1984). Effects of intraaortic balloon counterpulsation on myocardial blood flow in patients with severe coronary artery disease. J. Am. Coll. Cardiol..

[28] Fuchs R.M., Brin K.P., Brinker J.A., Guzman P.A., Heuser R.R., Yin F.C. (1983). Augmentation of regional coronary blood flow by intra-aortic balloon counterpulsation in patients with unstable angina. Circulation.

[29] Patel M.R., Smalling R.W., Thiele H., Barnhart H.X., Zhou Y., Chandra P., Chew D., Cohen M., French J., Perera D. (2011). Intra-aortic Balloon Counterpulsation and Infarct Size in Patients with Acute Anterior Myocardial Infarction Without Shock: The CRISP AMI Randomized Trial. JAMA.

[30] Curtis J.P., Rathore S.S., Wang Y., Chen J., Nallomothu B.K., Krumholz H.M. (2012). Use and effectiveness of intra-aortic balloon pumps among patients undergoing high risk percutaneous coronary intervention: Insights from the National Cardiovascular Data Registry. Circ Cardiovasc. Qual Outcomes.

[31] Briceno N., Kapur N.K., Perera D. (2016). Percutaneous mechanical circulatory support: Current concepts and future directions. Heart.

[32] Kirtane A.J., Doshi D., Leon M.B., Lasala J.M., Ohman E.M., O’Neill W.W., Shroff A., Cohen M.G., Palacios I.F., Beohar N. (2016). Treatment of higher-risk patients with an indication for revascularization: Evolution within the field of contemporary percutaneous coronary intervention. Circulation.

[33] McCabe J.M. (2018). Hemodynamic Support for CTO PCI: Who, When, & How.

[34] van Diepen S., Katz J.N., Albert N.M., Hery T.D., Jacobs A.K., Kapur N.K., Kilic A., Menon V., Ohman E.M., Sweitzer N.K. (2017). Contemporary management of cardiogenic shock: A scientific statement from the American Heart Association. Circulation.

[35] Papaioannou T.G., Terrovitis J., Kanakakis J., Stamatelopoulos K.S., Protogerou A.D., Lekakis J.P., Nanas J., Stamatelopoulos S. (2002). Heart rate effect on hemodynamics during mechanical assistance by the intra-aortic balloon pump. Int. J. Artif. Organs.

[36] O’Brien J., Reid C.M., Andrianopoulos N., Ajani A.E., Clark D.J., Krum H., Loane P., Freeman M., Sebastian M., Brennan A.L. (2018). Heart Rate as a Predictor of Outcome Following Percutaneous Coronary Intervention. Am. J. Cardiol..

[37] Gaggin H.K., Januzzi J.L. (2013). Biomarkers and diagnostics in heart failure. Biochim. Biophys. Acta (BBA)—Mol. Basis Dis..

